# Thyroid incidentalomas with increased focal ^18^F-FDG uptake in ^18^F-FDG PET/CT of a patient with multiple primary cancers.

**DOI:** 10.1007/s12020-021-02661-w

**Published:** 2021-04-27

**Authors:** Patrick W. Mihatsch, Matthias Beissert, Thorsten A. Bley, Andreas K. Buck, Constantin Lapa

**Affiliations:** 1 Department of Diagnostic and Interventional Radiology, University Hospital of Würzburg, Würzburg, Germany; 2 Department of Nuclear Medicine, University Hospital of Würzburg, Würzburg, Germany; 3 Nuclear Medicine, Medical Faculty, University of Augsburg, Augsburg, Germany

**Fig. 1:**
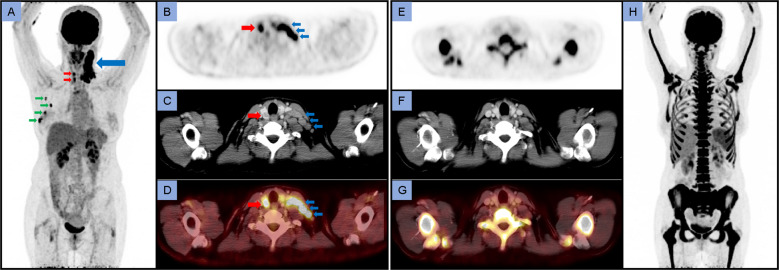
A 54-year old woman with newly diagnosed EBV-positive classical Hodgkin’s lymphoma was referred for initial staging with ^18^F-FDG PET/CT. The ^18^F-FDG PET/CT scan revealed a conglomerate of multiple hypermetabolic cervical nodal manifestations on the left side (level II–V, Deauville score 5, SUV_max_ 40.7), corresponding to a stage II disease, as shown on the maximum intensity projection image (**A**; blue arrow). In addition, multifocal intense ^18^F-FDG uptake in the right breast (SUV_max_ 14.4) and hypermetabolic axillary lymph nodes on the right side (SUV_max_ 11.3) — highly suspicious for a second primary malignancy — were detected (**A**; green arrows). Consecutive fine-needle aspiration cytology of all lesions confirmed the simultaneous diagnosis of multifocal breast cancer (G2, HER2-positive) with axillary metastases on the right side. Moreover, the initial ^18^F-FDG PET/CT scan revealed two focal lesions in the right thyroid lobe (SUV_max_ 11.0; **A**–**D**; red arrows). Given the confirmed diagnosis of two tumor entities and the lack of a therapeutic consequence, initial histopathological examination of the two thyroidal lesions was not performed. After three cycles of neoadjuvant chemotherapy (3 × 5-fluorouracil, epirubicin, and cyclophosphamide (FEC); 3 × docetaxel, trastuzumab, and pertuzumab) and additional radiation therapy to the neck, the patient received a follow-up ^18^F-FDG PET/CT scan that revealed a complete response both of the Hodgkin’s lymphoma (according to Lugano 2014 criteria) and the metastasized breast cancer (in terms of RECIST and PERCIST) with concomitant reactive activation of the bone marrow and of the spleen (**H**). Interestingly, the two previously hypermetabolic thyroid lesions also showed a complete response (**E**–**G**) — ultimately indicating a malignant origin, e.g., Hodgkin’s lymphoma of the thyroid, breast cancer metastases to the thyroid gland or a third primary thyroid tumor. While the incidental finding of a focal thyroid ^18^F-FDG uptake in ^18^F-FDG PET/CT is rare and only occurs at a frequency of 1.1–4.2% [1], thyroid incidentalomas carry a significant risk of malignancy that is reported to be 23.0–63.6% [1]. This risk of malignancy is especially high when thyroid lesions show focal ^18^F-FDG uptake [2, 3], i.e., when the PET scan (rather than the CT image) shows a suspicious finding and when their SUV_max_ is above 4.2 [2]. Histopathological evaluation of thyroid incidentalomas shows papillary thyroid carcinoma to be the most prevalent thyroid malignancy, whereas metastases to the thyroid gland are mostly derived from renal cell carcinoma (in a clinical setting) or lung cancer (in autopsy series). Hodgkin’s lymphoma of the thyroid shows a female preponderance, but is extremely rare, and breast cancer metastases to the thyroid are seldomly reported. However, an association between thyroid cancer and breast cancer has been described in the literature. For the evaluation of a thyroid incidentaloma, both PET (focal ^18^F-FDG uptake, high SUV_max_) and CT (low attenuation) can be helpful [2] while ultrasound is still the mainstay to stratify the risk of malignancy. Still, prompt histopathological examination should be performed for definitive diagnosis. Here, a biopsy of the thyroid incidentalomas would have been obligatory in case of persistence or progression under treatment

## Data Availability

Relevant documentation or data in order to verify the validity of the results presented will be provided upon request.
